# Estimating effects of monsoon flooding on household water access

**DOI:** 10.1088/1748-9326/ad6ce9

**Published:** 2024-08-16

**Authors:** Lauren M T Broyles, Emily L Pakhtigian, Alfonso Mejia

**Affiliations:** 1 Population Research Institute, The Pennsylvania State University, 601 Oswald Tower, University Park, PA 16802, United States of America; 2 School of Public Policy, The Pennsylvania State University, 322 Pond Laboratory, University Park, PA 16802, United States of America; 3 Civil and Environmental Engineering, The Pennsylvania State University, 212 Sackett Building, University Park, PA 16802, United States of America

**Keywords:** climate change, extreme weather event, monsoon flooding, Bangladesh, household water access

## Abstract

The importance of climate in water resources management is well recognized, but less is known about how climate affects water access at the household level. Understanding this is crucial for identifying vulnerable households, reducing health and well-being risks, and finding equitable solutions. Using difference-in-differences regression analyses and relying on temporal variation in interview timing from multiple, cross-sectional surveys, we examine the effects of monsoon riverine flooding on household water access among 34 000 households in Bangladesh in 2011 and 2014. We compare water access, a combined measure of both water source and time for collection, among households living in flood-affected and non-flood-affected districts before and after monsoon flooding events. We find that households in monsoon flood-affected districts surveyed after the flooding had between 2.27 and 4.42 times higher odds of experiencing low water access. Separating geographically, we find that while households in coastal districts have lower water access than those in non-coastal districts, monsoon flood exposure is a stronger predictor of low water access in non-coastal districts. Non-coastal districts were particularly burdened in 2014, when households affected by monsoon flooding had 4.71 times higher odds of low water access. We also find that household wealth is a consistent predictor of household water access. Overall, our results show that monsoon flooding is associated with a higher prevalence of low water access; socioeconomically vulnerable households are especially burdened.

## 1. Introduction

Climate change is expected to increase pressure on water resources by increasing frequency of extreme weather events and shifts in the timing of and variability in rainfall and temperature (IPCC 2022). Further, some of the world’s most vulnerable populations will bear disproportionate burdens of climate change impacts (Levy and Patz 2015). Already, four billion people experience severe water scarcity for at least part of the year (Mekonnen and Hoekstra 2016), and 2.4 billion people lack safely managed drinking water (UN 2023). Increased pressure on global water resources will reduce water availability, increasing water insecurity and exacerbating consequences for health and well-being (Mukherji 2022). The Global Commission on the Economics of Water (GCEW) recently drew attention to the urgency of this problem through its call for a mission-driven approach to addressing global water insecurity (GCEW 2023, Rockström *et al* 2023). To respond to this global water challenge, we must develop a holistic and nuanced understanding of how shifts in climate and extreme weather events affect household water access.

Bangladesh is especially vulnerable to the water-related impacts of climate change (Chowdhury *et al* 2022). While over 80% of Bangladeshi households have water sources at their homes (UN Water 2024), annual monsoon flooding displaces hundreds of thousands of people and negatively affects food and water access (NAWG 2020, UN Bangladesh 2023). Accordingly, extreme weather events and other climatic factors may contribute to household water insecurity, a condition when problems with water, such as reduced water access, affordability, reliability, and safety, negatively affect overall health and well-being (Jepson *et al* 2017). There is well-documented literature that examines how groundwater salinity, surface water contamination, and arsenic contamination threaten water safety in Bangladesh (see, for example, Akanda *et al* 2013, Benneyworth *et al* 2016, and Bhattacharya *et al* 2011). Further, seasonality in Bangladesh contributes to periodic water scarcity, particularly in the months prior to the monsoon (Hoque 2021, Broyles *et al* 2023). While flooding has been shown to negatively affect migration (Giannelli and Canessa 2022), food security (Smith and Frankenberger 2018, Parvez *et al* 2022), and health (Rerolle *et al* 2023), associations with water security have not been studied. In other contexts, such as the Bolivian Amazon and Tanzania, studies find that flooding can increase water insecurity through degrading water quality and destroying water source infrastructure (Rosinger 2018, Smiley and Hambati 2019). To fill these literature gaps, we examine the short-term effects of monsoon flooding on household water access—a combined measure of water source and time (Howard *et al* 2020)—in Bangladesh. We focus on water access as one dimension of water insecurity due to the lack of historical country-wide household water insecurity data based on an experiential scale (e.g. HWISE and IWISE (Young *et al* 2019, 2021a, 2021b, Stoler *et al* 2021, Bethancourt *et al* 2022)). We further examine how geography and socioeconomic status moderate this relationship.

We use historical flood and weather (temperature and precipitation) data and household surveys to estimate the effects of monsoon flooding on household water access across Bangladesh. Bangladesh provides a distinctive setting for this analysis due to its vulnerability to climate change impacts related to flooding, cyclones and storm surges, salinity intrusion into groundwater, temperature extremes, and moisture stress during dry periods (Huq and Ayers 2008, Eckstein *et al* 2021, Getirana *et al* 2022, The World Bank Group 2024). While focused on Bangladesh, our analysis may provide broader insight for water management—especially in countries with monsoon climates that face water developmental challenges—while also responding to the need for increased understanding of seasonal and climate effects on household water access (Broyles *et al* 2022).

To test the effects of flood exposure on household water access we examine two different monsoon flooding events—one each in 2011 and 2014. While flooding during both years was typical of the monsoon season, flooding in 2014 was more severe (FFWC & BWDB 2011, 2014). In 2011, moderate flooding began in August in the northern and central parts of Bangladesh and prolonged flooding began in the southwest. During the 2014 monsoon, flooding was concentrated in August and September and was particularly severe in the northeastern part of the country. Despite past and ongoing flood adaptation and mitigation efforts in Bangladesh (Rahman and Salehin 2013, Rahman 2022), monsoon flooding routinely damages infrastructure, brings property and livestock loss, diminishes food security, and threatens economic stability, especially for the most vulnerable (FFWC, & BWDB 2011). For the analysis, we use data from the Bangladesh Demographic and Health Survey (DHS), the Indian Monsoon Data Assimilation and Analysis (IMDAA) project (Rani *et al* 2021), and the EM-DAT Disaster Database (CRED 2023). For each flood event, we exploit variation in the timing of cross-sectional survey data collection and monsoon flooding and use difference-in-differences models to estimate the effects of monsoon flooding on household water access, comparing outcomes among households living in flood-affected and non-flood-affected areas before and after the flooding events. To assess the validity of our difference-in-differences designs and provide insight into the dynamic effects of flooding, we estimate event study analyses and conduct robustness checks using matched samples. We further test for heterogeneity in effects based on geography (coastal versus non-coastal) and socioeconomic status (low versus high wealth).

## 2. Methods

### 2.1. Data

We use data from the Bangladesh DHS, the IMDAA project, and the EM-DAT disaster database. We use the Bangladesh DHS Persons Recode files from the 2011 and 2014 waves, which are nationally representative and contain 17 141 and 17 300 households, respectively (NIPORT *et al* 2013, 2016). These data were collected between July and December in 2011 and June and November in 2014, which coincide with the pre-monsoon, monsoon, post-monsoon, and dry seasons in Bangladesh. Data were collected in parallel across the country, generating temporal and geographical variation within each wave. We use the 2011 and 2014 DHS waves to exploit timing of survey data collection prior to and following monsoon flood events and to allow for consistency in the definition of our main outcome of interest—low water access. We conduct all empirical analyses with Stata/SE 18.0 and generate nationally representative estimates using corresponding survey weights and Stata *svy* commands (StataCorp 2023a, 2023b).

The DHS datasets contain social and demographic variables along with household water use information. They also include geographic information for the DHS sampling unit (cluster), which contain an average of 30 households. In the dataset, cluster coordinates are offset to preserve anonymity of surveyed households. The offset is up to 2 km in urban areas and 5 km in rural areas; 1% of the rural clusters are displaced up to 10 km (Burgert *et al* 2013). Across all variables used in the analysis, less than 1% of observations have missing data.

We use the cluster GPS coordinates to extract historical temperature and precipitation data from the IMDAA project from 2008 to 2018, which we use to calculate climatic averages (Rani *et al* 2021). We extract hourly mean, minimum, and maximum temperatures (C) and hourly mean accumulated precipitation (mm). We develop 10 year climate averages for mean, minimum, and maximum temperatures and accumulated precipitation for each cluster. Based on these 10 year climate averages, we calculate the percentage difference between the accumulated precipitation during the DHS survey month and the climate average for that month for each cluster to generate point-by-point estimates of the weather and climate for each cluster.

We use the EM-DAT disaster database, which is maintained by the Centre for Research on the Epidemiology of Disasters (CRED) to determine which districts were affected by seasonal monsoon flooding in 2011 and 2014 (CRED 2023). The EM-DAT uses a broad set of sources, such as the United Nations, research institutes, insurance companies, government and non-government agencies, and the press, to identify areas affected by flooding. While the EM-DAT reports both riverine and flash floods, we use only riverine flood events due to the different hydrological processes that generate these flooding events. We further validate riverine monsoon flooding for both years using government-level reports (FFWC, & BWDB 2011, 2014). Separately for 2011 and 2014, we classify households as flood-affected if the household was interviewed following the onset of monsoon flooding and located in a flood-affected district per the EM-DAT disaster database classification. Importantly, we assess flood exposure as flood-affected is defined at a geographical (district) level, rather than based on individual household’s inundation experiences.

### 2.2. Water access outcome variable

We use data on household water source and water collection time to create our primary outcome of interest—low water access. Similar to other studies (Brewis *et al* 2019, Choudhary *et al* 2020), we define water access according to World Health Organization guidelines (Howard *et al* 2020). We rely on water source and time for water collection as they are available in both the 2011 and 2014 DHS waves. Water access levels include optimal (water available in the house), intermediate (maximum 5 min to fetch), and low (collapsing basic and no access WHO categories due to lack of data for differentiation). We create an indicator for low water access, which takes a value of 1 for households in the low access category. Further details related to this classification are available in supplementary table 1.

### 2.3. Control variables

We use household size, household head age, household head gender, household head education, a household asset index, and a rural indicator as control variables in our analysis. We include household size, household head age, household head gender, household head education, and household assets as other studies have shown them to affect household water access (Morakinyo *et al* 2015, Belay and Andualem 2022). We develop our own household asset index because the wealth index created by the DHS includes water source variables (USAID 2023). To generate the asset index, we use the first component of a principal component analysis (Stata *pca* command), which was estimated using ownership of radio, television, mobile phone, non-mobile phone, refrigerator, bank account, bicycle, motorcycle; household flooring, roof, and wall material; and access to electricity.

### 2.4. Empirical method

#### 2.4.1. Difference-in-differences specification

To estimate the effects of monsoon flooding on household water access, we use a difference-in-differences regression specification in which we compare household water access among households in flood-affected districts with those in non-flood-affected districts prior to and following the onset of monsoon flooding in 2011 and 2014. We estimate the following logistic fixed effects model separately for 2011 and 2014:

\begin{align*}{\text{Wate}}{{\text{r}}_{ijt}} &amp; = {B_0} + {B_1}{X_{ijt}} + {B_2}{F_{ij}} + {B_3}{\text{Pos}}{{\text{t}}_t} + {B_4}\left( {{F_{ij}}x{\text{Pos}}{{\text{t}}_t}} \right)\nonumber\\ &amp;\quad + { }{\alpha _j} + {\varepsilon _{ijt}}.\end{align*}

Here, ${\text{Wate}}{{\text{r}}_{ijt}}$ is an indicator for low water access for household *i* in cluster *j* at month *t.* ${X_{ijt}}$ is a vector household and cluster controls. At the household level, these include household size, household head education level, household head age, household head gender, and household asset index. At the cluster level, these variables include whether the cluster is in a rural or urban area, the maximum temperature recorded for the month in which the household survey was conducted, and the percentage difference between the precipitation in the month in which the survey is conducted and climate averages for that month. ${F_{ij}}$ is an indicator variable for location in a flood-affected district. ${\text{Pos}}{{\text{t}}_t}$ is an indicator variable for being surveyed after the flood occurred. The interaction term ${F_{ij}}x{\text{Pos}}{{\text{t}}_t}$ identifies households that were surveyed after the onset of monsoon flooding in flood-affected areas. ${\alpha _j}$ is province-level fixed effect. ${\varepsilon _{ijt}}$ is our error term. Standard errors are estimated according to Taylor linearization, the *svyset* default estimate for standard errors within survey design (Wolter 2007, StataCorp 2023b). This method of standard error estimation is equivalent to the robust variance estimator for non-survey data analysis.

#### 2.4.2. Event study specification

To investigate the validity of our difference-in-differences estimates and to explore the dynamic effects of flooding on household water access, we estimate the following event study specification separately for 2011 and 2014:

\begin{align*}{\text{Wate}}{{\text{r}}_{ijt}} &amp; = \mathop \sum \limits_{z = - 2,{ }z \ne - 1}^3 \left[ {{\pi _z}*{F_{ij}}*1{ }\left( {t - {\text{month}} = z} \right)} \right]\nonumber\\ &amp;\qquad + {B_1}X{1_{ijt}} + { }{B_2}{F_{ij}} + {\text{Mont}}{{\text{h}}_t} + {\varepsilon _{ijt}}.\end{align*}

All components of equation (2) are the same as in equation (1), although we interact ${F_{ij}}$ with event-time dummies. For 2011, the sequence of ${\pi _z}$ coefficients for $z = - 1, \ldots ,4{\text{ }}\left( {z = 0{\text{ omitted}}} \right)$ traces out one month before the onset of monsoon flooding and 4 months following. For 2014, the sequence of ${\pi _z}$ coefficients for $z = - 2, \ldots ,3{\text{ }}\left( {z = 0{\text{ omitted}}} \right)$ traces out two months before the onset of monsoon flooding and 3 months following. We include a monthly fixed effect (${\text{Mont}}{{\text{h}}_t})$.

#### 2.4.3. Heterogeneity analyses

We estimate heterogeneity in the effects of monsoon flooding on household water access by geography and socioeconomic status. To assess differential effects by geography, we classify districts as coastal or non-coastal according to the coastal regions listed in Uddin and Kaudstaal (2003) and re-estimate equations (1) and (2) on each geographical subsample. Coastal and non-coastal areas have different environmental vulnerabilities; for example, coastal areas in Bangladesh experience different environmental vulnerabilities, such as cyclones and storm surge (Mallick *et al* 2011). To assess differential effects by socioeconomic status, we classify households as high and low wealth based on their asset index falling above (high) or below (low) the median asset index in the sample and re-estimate equations (1) and (2) on each socioeconomic subsample.

#### 2.4.4. Robustness checks

We conduct three sets of robustness checks related to our analytical approach and other water quality concerns in Bangladesh. First, as the validity of the difference-in-differences model relies on linear parallel trends, we re-estimate equation (1) using linear regression. Second, given potential differences between flood affected and non-affected households, we use propensity score matching (Stata *psmatch2* command) to construct matched samples that are more balanced on observable characteristics. We match households based on household asset index, rural/urban location, household size, household head education level, household head age, household head sex, water treatment, and province. We use 2 nearest neighbors and 1 nearest neighbor for matching. We re-estimate equation (1) using these matched samples.

Third, given the presence of arsenic contamination in some aquifers in Bangladesh, we re-estimate equation (1) controlling for high arsenic contamination. We match a geo-referenced historical map of groundwater arsenic concentrations (BGS & DPHE 2001) to DHS cluster locations to extract arsenic concentrations for all households in the 2011 and 2014 DHS surveys (supplementary figure 1). As these concentrations are coarsely defined, we control for high arsenic contamination using an indicator for concentrations at or above 50 *μ*g l^−1^ of arsenic (the Bangladesh drinking water standard for arsenic (Jameel *et al* 2021)). Controlling for arsenic contamination is an important consideration as households may switch away from typically higher quality water sources, such as tubewells, if they are known to be contaminated with arsenic (Goel *et al* 2020).

## 3. Results

We first present descriptive statistics related to water use and other household characteristics of our sample (supplementary table 2) and then our regression estimates. Descriptively, in both 2011 and 2014, over 85% of households obtained their drinking water from tubewells. Further, most households used water sources in their own yard or plot, and the average time required for collection of water was between 5 and 6 min, suggesting a limited travel burden associated with water collection. Aggregating these water-related variables, approximately 20%–23% of households have low water access. We observe differences between coastal and non-coastal areas. Households in coastal areas have longer average water collection times and higher reliance on surface water compared to households in non-coastal areas. Further, households in coastal areas have lower water access compared to non-coastal areas, with 40%–44% of coastal households having low water access, compared to 14%–15% of non-coastal households. The average household in our sample has between 4 and 5 members and over 70% of the sample is rural. The majority of households have middle aged, male household heads with a primary or lower education level.

### 3.1. Difference-in-differences estimates

**Figure 1.**
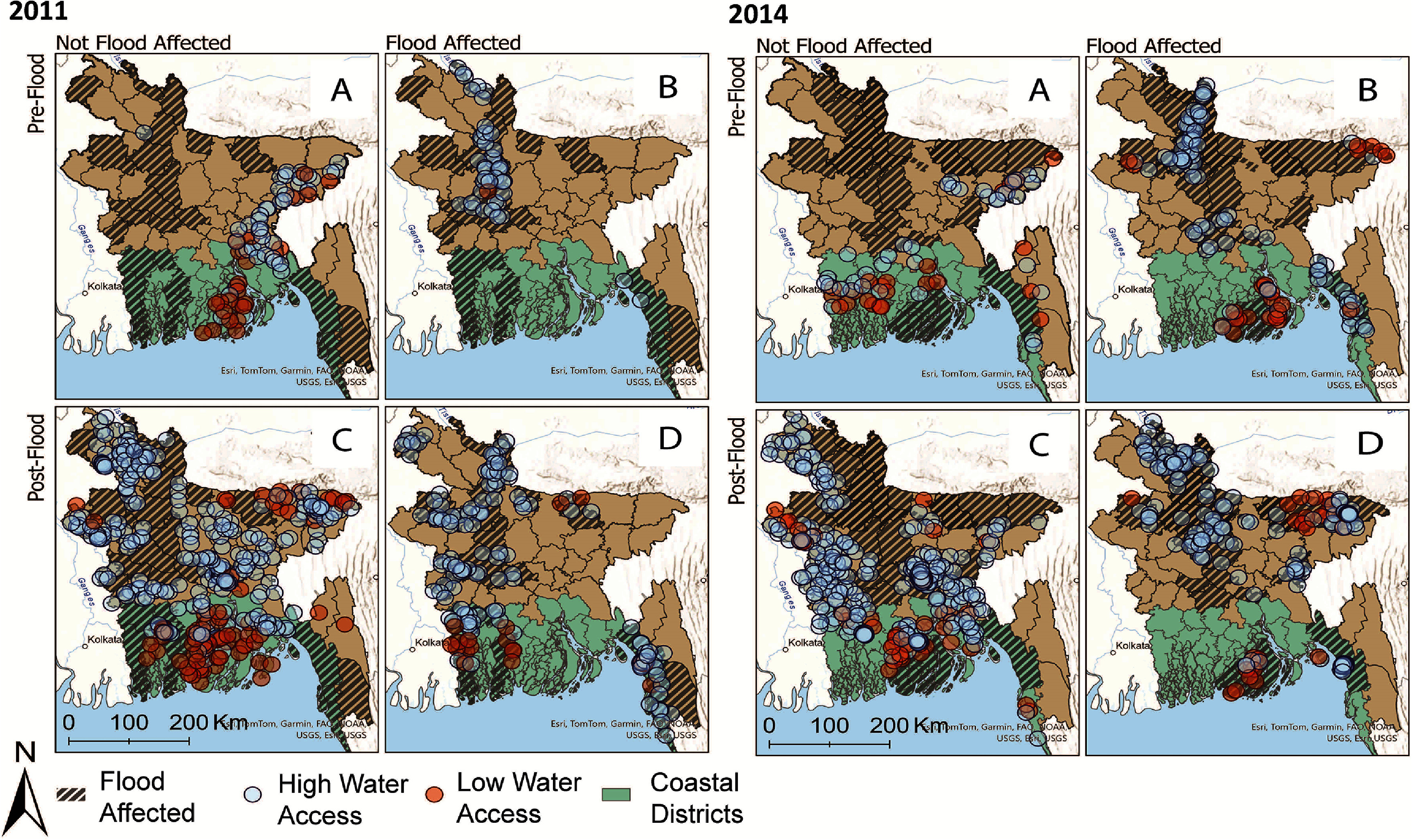
We use district-level flood exposure from the EM-DAT disaster database to identify which households from the DHS were exposed to monsoon flooding in 2011 and 2014. We validate district-level flood exposure and timing of floods with Bangladesh government reports (FFWC, & BWDB 2011, 2014). We use variation in survey timing and flood exposure to identify four groups of households, which are represented in the four panels in the above figure for each year: (A) households surveyed before the flood and not living in flood affected districts; (B) households surveyed before the flood and living in flood affected districts; (C) households surveyed after the flood and not living in flood affected districts; and (D) households surveyed after the flood and living in flood affected districts. Households in panel (D) represent our treated group for the difference-in-differences models. Each panel shows prevalence of low water access at the DHS cluster level. DHS clusters were identified as having low water access if more households within the cluster were coded as having low water access compared to households that were identified as having intermediate or optimal water access. We also examine heterogeneity by coastal status, which is identified by the green shading in the figure.

Figure 1 depicts a visual representation of our regression estimation, the results of which we present in table 1 (full regression results are presented in supplementary table 3). Results from the difference-in-differences models show that in both 2011 and 2014, households in monsoon flood-affected areas interviewed during or following the flood events have higher odds of experiencing low water access. This means that these households are more likely to rely on lower quality water sources and/or spend more than 5 min on daily water collection. The magnitude of the effect size varies somewhat between 2011 and 2014. In 2011, flood affected households had 4.42 times higher odds of experiencing low water access (*p* < 0.001, 95% CI: 2.23, 8.76) and, in 2014, flood affected households had 2.27 higher odds of experiencing low water access (*p* < 0.05, 95% CI: 1.19, 4.32). While the monsoon flooding events in 2011 and 2014 differed, making it difficult to directly compare these estimates, it is consistent that we might expect larger effects in 2011. For example, low household water access was more widespread in 2011 compared to 2014. Accordingly, with limited alternatives, the consequences of flooding on water access may have been more severe in 2011. In addition to our primary model, we estimate our difference-in-differences specification with province fixed effects to account for time-invariant regional differences. Including these fixed effects, our results are descriptively consistent, although less precisely estimated in some cases. In 2011, the estimated effect is positive; however, the coefficient is no longer significant as the confidence interval for the odds ratio includes 1. In 2014, we find that households affected by monsoon flooding have 2.94 times the odds of experiencing low water access (*p* < 0.01, 95% CI:1.51, 5.76).

**Table 1.** Effects of monsoon flooding on household water access.

	2011				2014			
	1		2		3		4	
***Panel (A): Entire country***								

	Low water access		Low water access (province FE)		Low water access		Low water access (province FE)	
	Odds ratio (linear. SE)	95% CI	Odds ratio (linear. SE)	95%	Odds ratio (linear. SE)	95% CI	Odds ratio (linear. SE)	95% CI

Post*Flood	4.42 *** (1.54)	**[2.23, 8.76]**	1.18 (0.41)	[0.60, 2.33]	2.27 * (0.75)	**[1.19, 4.32]**	2.94 ** (1.01)	**[1.51, 5.76]**

*N*	17 089		17 060		17 079		17 001	

***Panel (B): Coastal districts***								

	Low water access		Low water access (province FE)		Low water access		Low water access (province FE)	
	Odds ratio (linear. SE)	95% CI	Odds ratio (linear. SE)	95% CI	Odds ratio (linear. SE)	95% CI	Odds ratio (linear. SE)	95% CI

Post*Flood	2.11 (1.28)	[0.64, 6.96]	1.45 (0.88)	[0.44, 4.78]	1.32 (0.54)	[0.59, 2.93]	1.83 (0.79)	[0.79, 4.28]

*N*	5449		5449		5239		5239	

***Panel (C): Non-coastal districts***								

	Low water access		Low water access (Province FE)		Low water access		Low water access (province FE)	
	Odds ratio (linear. SE)	95% CI	Odds ratio (linear. SE)	95% CI	Odds ratio (linear. SE)	95% CI	Odds ratio (linear. SE)	95% CI

Post*Flood	2.10 (0.89)	[0.91, 4.82]	1.22 (0.53)	[0.52, 2.89]	4.71 *** (2.18)	**[1.90, 11.69]**	1.62 (0.79)	[0.62, 4.24]

*N*	11 640		11 611		11 840		11 762	

*notes*: *** *p* < 0.001, ** *p* < 0.01, * *p* < 0.05 Analysis conducted with DHS survey weights. Results reported as odds ratio (linearized std. error) with 95% CI. Standard errors are estimated according to Taylor linearization. Other controls included are survey month maximum temperature, difference between monthly accumulated precipitation from climate average, whether household was in rural or urban location, asset index, household size, household head education, household head gender, and household head age. Columns 1 and 3 use models without province fixed-effects, and columns 2 and 4 use province-fixed effects.

We assess geographical heterogeneity by estimating our difference-in-differences specification for coastal and non-coastal districts (table 1). We find that the increased reliance on low water access associated with monsoon flooding seems to be driven by households in non-coastal districts, particularly in 2014 (table 1). Flood-affected households in non-coastal districts had 2.10–4.71 times the odds of needing to rely on low water access (*p* < 0.001, 95% CI: 1.90, 11.69) compared to those that were not flood-affected.

Across the difference-in-differences models, we find that households with higher asset indices have lower odds of relying on low water access (supplementary table 3). Thus, even after controlling for seasonality and flood exposure, poorer households consistently have higher odds of using poor quality water sources or relying on sources farther from their homes. To assess whether wealth moderates the effect of flooding on household water access, we conduct a heterogeneity analysis by socioeconomic status (supplementary table 4). In general, we find that the effects of flooding on household water access are more damaging and more precisely estimated among low wealth households. This socioeconomic dimension of water access reinforces a crucial point—the most vulnerable households are at greatest risk from climate-related events, as evidenced by the case of monsoon flooding.

### 3.2. Event study estimates

**Figure 2.**
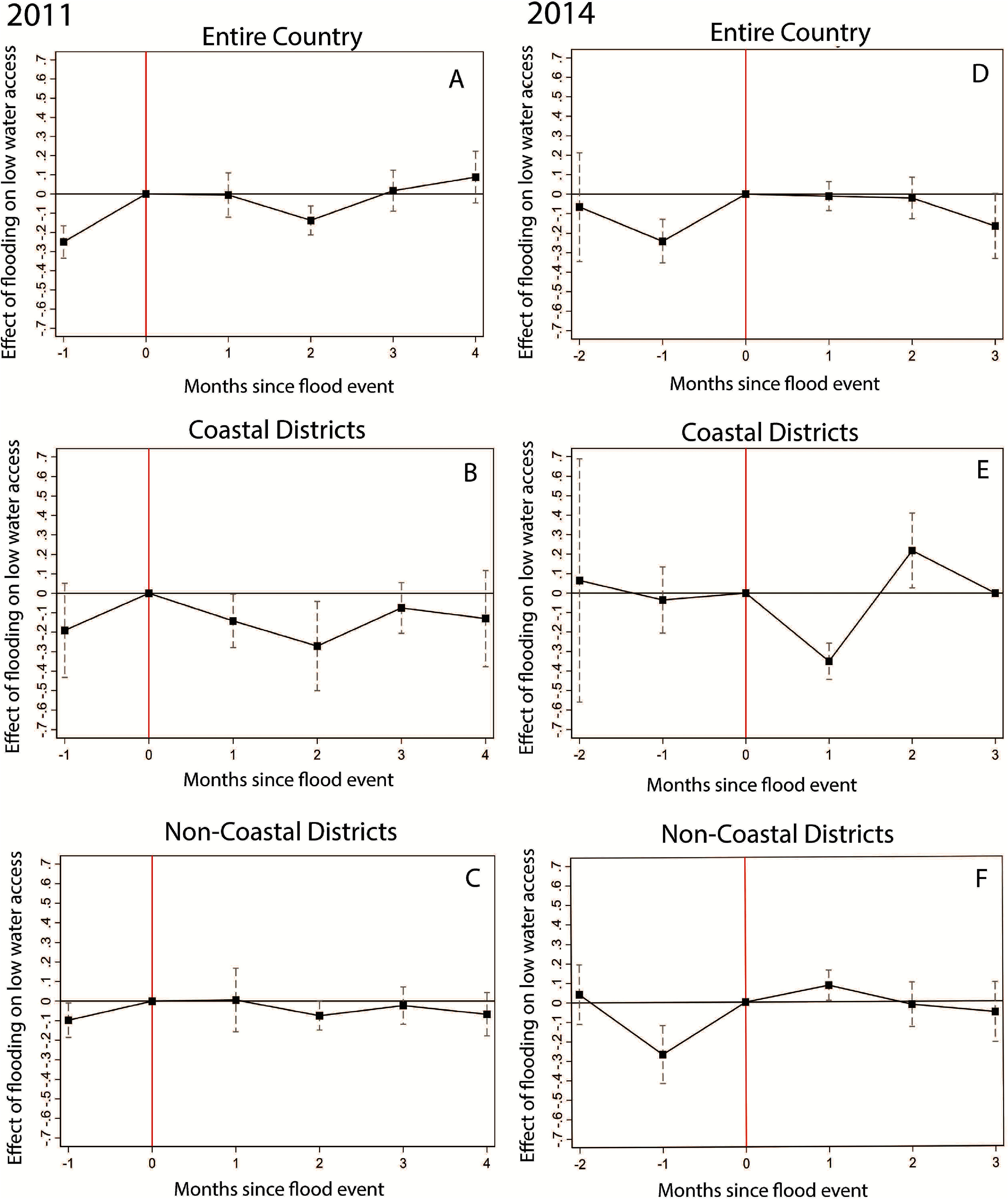
Event study coefficients showing the dynamic effects of flood exposure on low water access in the months before and after monsoon flooding (onset of monsoon flooding (Month 0 = August) omitted). We conduct this analysis for the entire country as well as for coastal and non-coastal districts for both 2011 and 2014. Panels (A), (B) and (C) reflect the entire country, coastal districts, and non-coastal district analyses for 2011. Panels (D), (E) and (F) reflect the entire country, coastal districts, and non-coastal district analyses for 2014. In 2011, Month −1 = July indicates one month before onset of monsoon flooding, and Month 1 = September through Month 4 = December indicate the four months after monsoon flooding began. In 2014, Month −2 = June and Month −1 = July indicate the two months before onset of monsoon flooding, and Month 1 = September through Month 3 = November indicate the three months after monsoon flooding began. Coefficient estimates are reported in supplementary tables 5 and 6.

To examine the dynamic effects of monsoon flooding on low water access, we conduct event study analyses (figure 2 plots the event study coefficients, and supplementary tables 5 and 6 present the event study regression results). For 2011, we see evidence of differential low water access between flood-affected and non-flood affected households in the month before the onset of monsoon flooding; low water access is significantly less prevalent in flood-affected districts in the overall sample (Panel (A): Coeff: −0.25, *p* < 0.0001, 95% CI: −0.33, −0.17) and in coastal and non-coastal district subsamples (Panels (B) and (C)). Immediately following the onset of monsoon flooding, there is no measurable difference in water access between flood-affected and non-flood-affected households in the full sample; low household water access decreases among flood affected households in coastal districts in the month following the start of the floods. Two months following the onset of monsoon flooding, lower water access decreases among flood-affected households, particularly in coastal districts. Finally, by three and four months following the onset of monsoon flooding, our estimates are insignificant, suggesting that impacts are not sustained. While it may be somewhat unexpected that water access improves in coastal districts one and two months after the onset of the monsoon flooding (Panel (B)), this could be due to survey sampling and households surveyed in these months were largely from the Chittagong district, which had overall higher water access and, thus, more potential for water switching to high water access sources.

For 2014, we find that prevalence of low water access is generally lower before the flood, with significant differences for the entire country and non-coastal differences in the month preceding the flooding (Panel (D): Coeff: −0.24, *p* < 0.001, 95% CI: −0.35, −0.13, Panel (F): Coeff: −0.27, *p* < 0.001, 95% CI: −0.42, −0.12). For our country-level estimates, we find no difference in low water access between flood-affected and non-flood-affected households in the three months following the onset of flooding. In coastal areas, low water access decreases among flood-affected households one month after the flood but then increases in the second month (Panel (E): September*Flood Coeff: −0.35, *p* < 0.001, 95% CI: −0.44, −0.26; October*Flood Coeff: 0.22, *p* < 0.05, 95% CI: 0.03, 0.41). This may be because flooding was most severe in non-coastal districts in the country, which could lead to lagged effects of flooding as water flows through Bangladesh into the Bay of Bengal. In non-coastal districts, however, one month after the flood, low water access is significantly higher among flood-affected households; this effect subsides two to three months following the onset of flooding (Panel (F): Coeff: 0.08, *p* < 0.05, 95% CI: 0.007, 0.16), again suggesting that the effects are not sustained. These dynamic patterns suggest that the severity of flooding, infrastructure available for flood mitigation and adaptation, and longevity of flooding impact may lead to different water access experiences among flood-affected and non-flood-affected households. Yet, the analysis points to concerns related to existing pre-trends and demonstrates the need for analysis over longer time periods.

### 3.3. Robustness checks

Given threats to identification and other contextual factors, we conduct three robustness checks. First, while we estimate our main specification using logit models and present our primary results as odds ratios because of our binary outcome, the parallel trends assumption that underlies identification via difference-in-differences relies on the linearity of estimation (Blundell and Dias 2009). Accordingly, we estimate our difference-in-differences model using linear regression (supplementary tables 7 and 8); the results which are consistent with our main findings.

Second, the event study analyses suggested the presence of pre-trends, a direct threat to identification. Further, the data from the 2011 and 2014 Bangladesh DHS waves were collected from July to December and June to November, respectively, while the monsoon flooding events primarily took place in August of each year. The survey and flooding timing results in limited pre-flood event data in each wave, constraining our ability to more fully evaluate pre-trends in our setting. Given these data limitations, we use propensity score matching with one nearest neighbor and two nearest neighbors to construct samples of flood-affected and non-flood-affected households that are more comparable (see balance tables supplementary tables 9 and 10). While the matched samples are observationally more similar, the matching process does not remove all differences in observables between households that do and do not experience monsoon flooding. Our difference-in-differences results using these matched samples are also consistent with our primary findings (supplementary tables 11 and 12). While the magnitudes of the results from the matched samples vary slightly, they communicate a consistent message that flood affected households have higher odds of low water access.

Third, naturally occurring groundwater arsenic contamination presents a significant water quality concern in Bangladesh, which may affect household water access. We re-estimate our primary models controlling for high groundwater arsenic concentrations. Controlling for high groundwater arsenic contamination, the results are consistent with our main findings (supplementary table 13). In 2011 and 2014, households interviewed during or after monsoon flooding in flood-affected districts had higher odds of low water access compared to households interviewed prior to flooding or in districts that were not flood affected. Further, non-coastal households were particularly affected in 2014.

## 4. Discussion

In this paper, we examine how monsoon flooding impacts water access across Bangladesh. We find that flood-affected households have lower water access, yet our dynamic models suggest that the impacts may not be sustained. Further, we find that while water access was generally lower in coastal districts, the negative impacts of flood exposure on water access are larger in non-coastal areas. In Bangladesh, water access is often more limited in coastal areas due to multiple causes, including a high water table that contributes to salty groundwater and may impede the installation of boreholes or other improved water systems; vulnerability to cyclones that can destroy water access points and other infrastructure; and construction of polders designed to protect against cyclones but also slow flood water drainage (Mauchamp *et al* 2002, Mallick *et al* 2011, Benneyworth *et al* 2016, Ali and Syfullah 2017). Despite lower water access in coastal areas, we find that monsoon flooding is more impactful on water access among households living in non-coastal areas. These findings are consistent with other work that has found non-coastal areas, particularly in northeastern Bangladesh, are more prone to flooding and its associated negative effects on household income, health, and educational outcomes (Giannelli and Canessa 2022, Rerolle *et al* 2023). This evidence suggests that flood events may make households more economically vulnerable, which could, in turn, reduce their resources available for water procurement. Further flood exposure may also directly affect water access through damage to infrastructure, such as damage to tubewell pumps or the flooding of shallow and deeper wells. While our analysis is unable to distinguish between the possible mechanisms underlying the association between monsoon flood exposure and water access, our findings suggest that flooding negatively impacts households’ abilities adequately access water for drinking and other domestic purposes.

While our study provides insight into how monsoon flooding affects water access, there are a number of important limitations and areas for future work to identify. First, households whose water points may have been most negatively affected by severe monsoon flooding may have chosen to leave prior to or following the flood event (Vestby *et al* 2024). It is estimated that over 400 000 people lived in temporary shelters due to monsoon flooding in 2011 (IFRC 2011) and 57 000 families were displaced between August and September of 2014 (IFRC 2014). Families that left or were displaced from flooding would be missing from the DHS survey data, likely leading to a conservative estimate of the effects of monsoon flooding on water access due to the omission of households most impacted by the flooding. Second, we used EM-DAT disaster data, which is at the districted level, to define flood exposure. Accordingly, we adopt a uniform flood-affected definition across households in each district and assume that flood impact was consistent across districts that experienced the same flood event. In practice, households who live closer to rivers or coastal areas may experience higher intensity of flooding. Incorporating satellite flood data (Giannelli and Canessa 2022, Rerolle *et al* 2023) could provide more precise estimates of flood effects on the prevalence of water access.

Third, we estimate effects on household water access, based only on the water source type and time for water collection, rather than a more comprehensive measure of household water insecurity. Our estimates do not capture the fuller household experience with their water source, which might also include dimensions such as financial cost for water collection, perceived water quality and safety, and reliability of the water source throughout the year, among others (Wutich and Ragsdale 2008, Bisung and Elliott 2018, Stoler *et al* 2020, Rosinger *et al* 2021, Broyles *et al* 2023). Water quality is of particular importance in Bangladesh due to well-known contaminants such as salinity, arsenic, water-borne pathogens, and the presence of other heavy metals (Bhattacharya *et al* 2011, Khan *et al* 2011, Akanda *et al* 2013, Benneyworth *et al* 2016). These other aspects of water insecurity may also be sensitive to the dynamic influence of weather variables throughout the year (Broyles *et al* 2022).

Fourth, while our setting is conducive to estimating differences in water access in districts that experienced and did not experience monsoon flooding prior to and following the onset of the floods, we are limited in our scope to examine the assumptions underlying the causal relationship between flood exposure and water access. Moreover, our event study estimates suggest there may be some differences in water access in flood affected and non-flood affected districts prior to the monsoon flooding in 2011 and 2014. Accordingly, we interpret our results as suggestive of negative consequences for water access resulting from monsoon flooding, yet we argue that additional analysis is needed to fully identify the causal relationship. Finally, Bangladesh experiences seasonal monsoon flooding as well as other extreme flood events. Given this context, the impacts of flooding on household water access may vary based on households’ historical flood experience, as households may learn and adapt over time (mitigating impact) or may experience compounding harms (exacerbating impact). While our data are unsuited to examine this heterogeneity, it would be instructive for future work to examine the cumulative impacts of flooding exposure on water access, and, where possible, include longitudinal analyses.

Through increased uncertainty in weather patterns and intensity and frequency of extreme events, climate change exerts additional stress on the global water system. Combined with unsustainable and inequitable management practices, this has led to negative social outcomes and a failure to meet basic human needs for water worldwide (Rockström *et al* 2023). For example, in Bangladesh and its neighbors, increasing groundwater use in agriculture combined with reduced rainfall at key points of the year has increased pressure on Bangladesh’s freshwater resources (Getirana *et al* 2022). Data from satellite analysis show that since 2000, water levels in some Bangladeshi aquifers have dropped by roughly one meter each year. This depletion of groundwater resources has spillover effects for household water security. Households must either exert more household resources towards pumping water from deeper ground wells or seek alternative water sources, which may be more expensive, further away, or of lower quality. Our analysis suggests that climate events, such as monsoon flooding, can further affect household wellbeing through impacts on water access.

## Data Availability

The data that support the findings of this study are openly available at the following URL/DOI: https://dhsprogram.com/; www.emdat.be/; https://rds.ncmrwf.gov.in/.
